# Clinical study on the screening of biomarkers for Crohn’s disease based on blood metabolomics

**DOI:** 10.3389/fgstr.2026.1867424

**Published:** 2026-07-23

**Authors:** Tongyuan Lin, Xianhong Hu, Kaifei Yuan, SiYuan Li, Ping Lin, Wei Wang, Li Cui, YuanYuan Wang, Ping Liu, Yong Yang

**Affiliations:** 1 The Department of Gastroenterology, The First People’s Hospital of Wu Hu, Wuhu, Anhui, China; 2 Postgraduate School, Anhui Normal University, Wuhu, Anhui, China; 3 The Department of Science and Education Section, The First People’s Hospital of Wu Hu, Wuhu, Anhui, China

**Keywords:** Crohn’s disease, high-performance liquid chromatography–tandem mass spectrometry, infliximab, metabolomics, ustekinumab

## Abstract

**Background:**

Crohn’s disease (CD) is a chronic inflammatory condition that affects the gastrointestinal tract. Currently, the diagnosis of CD is predominantly dependent on endoscopic procedures and histopathological tissue analysis. Endoscopy is an invasive procedure, patient compliance is poor, and the results are subject to the operator’s subjective judgment. Infliximab (IFX) and ustekinumab (UST) are two new biologics used for the treatment of CD, which are effective in reducing inflammatory markers. However, the correlation between blood metabolites and inflammatory markers has rarely been studied in the biologic agents employed in the management of CD, and their effects on metabolic processes remain inadequately understood. This study aimed to elucidate the effects of IFX and UST on the blood metabolites in patients with CD, investigate the correlations between metabolic variations and clinical indicators, and examine the relationship between the identified biomarkers and clinical efficacy.

**Methods:**

A total of 81 patients with CD who were receiving biologic therapy enrolled at week 16 between January 2022 and December 2023 (45 receiving IFX and 36 receiving UST) and 45 healthy controls were included. Metabolite profiling was performed using high-performance liquid chromatography–tandem mass spectrometry, followed by multivariate statistical and pathway enrichment analyses to identify differential metabolites and their correlations with clinical indicators.

**Result:**

Despite achieving clinical remission, both the IFX and UST groups exhibited significantly elevated C-reactive protein levels and erythrocyte sedimentation rate values compared with the control group (*p* < 0.05). IFX primarily influenced amino acid metabolism, including pathways involving arginine and glutamine, and glycolysis and the tricarboxylic acid cycle. UST impacted glutathione and purine metabolism. Pearson’s correlation analysis demonstrated that l-arginine, l-glutamine, and l-lysine were significantly negatively correlated with C-reactive protein. In the UST group, glutathione, adenosine, and hypoxanthine were primarily significantly negatively correlated with C-reactive protein and absolute lymphocyte count (*p* < 0.05).

**Conclusion:**

We identified two sets of diagnostic biomarkers: the biomarkers for the IFX group were l-arginine, l-glutamine, and l-lysine, while those for the UST group were glutathione and adenosine.

## 1 Introduction

Inflammatory bowel disease (IBD) is a chronic, recurrent, nonspecific inflammatory disease of the bowel that includes Crohn’s disease (CD) and ulcerative colitis (UC) (1). In recent years, the incidence of IBD has been increasing globally, particularly in Europe and North America. According to statistics, by 2025, more than 1.5 million people in China will have been affected by IBD, creating major economic and healthcare difficulties and thus making IBD a crucial public health challenge that demands urgent attention (2). Although the pathogenesis of CD remains unclear, it is thought to be driven by a complex interplay of genetic, environmental, immune, and microbial factors (3). Of these factors, microbial invasion has been shown in numerous studies to primarily trigger the release of pro-inflammatory cytokines, which in turn activate macrophages and subsequently induce the production of tumor necrosis factor alpha (TNF-α) (4–6). TNF-α plays a pivotal role in the pathogenesis of CD, with elevated levels of TNF-α leading to excessive inflammatory damage, which then initiates a cascade of aberrant and persistent immune responses (7, 8).

In the clinical management of CD, biologic therapies have shown promising therapeutic outcomes, with infliximab (IFX) and ustekinumab (UST) being the two widely utilized biologic agents. As outlined in the Chinese Expert Guide on Biologics for IBD, IFX, an anti-TNF-α novel biologic, is a chimeric human–mouse immunoglobulin G1 monoclonal antibody and was the first biologic approved for the treatment of CD (9). IFX functions by blocking both the soluble and transmembrane forms of TNF-α, thereby preventing its binding to receptors. The interaction of IFX with TNF-α results in the reduction of the cell adhesion molecule and chemokine levels, inhibiting the secretion of CD40/CD40L and the leukocyte infiltration into intestinal mucosal tissues (10). This process leads to a decrease in the serum high-sensitivity C-reactive protein (hs-CRP) levels and achieves an anti-inflammatory effect. Numerous studies have demonstrated that IFX can induce apoptosis to regulate the proliferation and migration of epithelial cells, activate extracellular signal-regulated kinase pathways to upregulate the expression of adhesion proteins, facilitate intestinal mucosal healing, and restore intestinal barrier function (11). UST is used in patients with CD who failed to respond to conventional drugs and IFX. UST is a fully human immunoglobulin G1κ (IgG1κ) monoclonal antibody targeting the p40 subunit, a shared component of interleukin-12 (IL-12) and interleukin-23 (IL-23) (12). IL-12 and IL-23 play crucial roles in Th1 and Th17 inflammatory responses. While Th1 and Th17 cells are essential for the maintenance of immune responses, their aberrant activation and differentiation can lead to the damage of intestinal mucosal immune cells. UST binds to the p40 subunit of IL-12 and IL-23, preventing these interleukins from interacting with the IL-12R-β1 receptor, thereby inhibiting downstream intracellular signaling and cytokine production and reducing the generation of Th1 and Th17 cells (13).

The diagnosis of CD currently relies predominantly on clinical manifestations, endoscopy, and histopathological examination. However, these methods present certain limitations. The initial symptoms of CD, which are primarily abdominal pain and diarrhea, complicate the achievement of an accurate diagnosis based solely on clinical presentation. Although endoscopy allows for direct observation of the changes in the intestinal lining, its invasive nature often results in poor patient compliance, and the findings are subject to various influences. Histopathological examination necessitates the collection of biopsy samples for microscopic analysis, a process that is both time-consuming and costly. In response to these limitations, researchers have initiated utilizing biomarkers for the diagnosis of CD. Metabolomics significantly contributes to the advancement of biomarker discovery and application. Using metabolomic approaches, researchers have identified that multiple metabolic pathways play significant roles in the pathogenesis of rheumatoid arthritis (RA), such as purine metabolism and glycerophospholipid metabolism (14). These findings provide a new explanation for the multi-target and multi-pathway anti-inflammatory mechanisms of RA.

Therefore, a cohort of 126 patients who visited the First People’s Hospital of Wuhu City (hereafter referred to as “our hospital”) between January 2022 and December 2023 were selected for this study. This population comprised a control group of 45 healthy individuals (*n* = 45) and an experimental group of 81 patients diagnosed with CD who have received medication treatment on the 16th week, which was further divided into an IFX group (*n* = 45) and a UST group (*n* = 36). Researchers collected data on the clinical manifestations, inflammatory indicators, biochemical factors, and Crohn’s Disease Activity Index (CDAI) scores of these patients. Blood samples from patients with CD and from healthy individuals were analyzed using high-performance liquid chromatography–tandem mass spectrometry (HPLC-MS/MS) to detect metabolite differences. Potential metabolic biomarkers were identified, and possible affected metabolic pathways were hypothesized.

## 2 Methods

### 2.1 Research population

This study is a single-center, exploratory, retrospective cohort analysis conducted at our hospital, which included 81 patients with CD who received biologic therapy between January 2022 and December 2023. In addition, 45 healthy individuals who underwent health examinations at the Health Examination Center of our hospital during this period were selected as the control group. For analytical purposes, the CD patients were classified into the remission and active phases based on comprehensive scoring criteria, including clinical symptoms, endoscopic examination, imaging results, and the CDAI scores (as detailed in **Supplementary Table 1**). Based on their intended treatment regimens, the patients were further divided into the UST group, with 18 cases in remission and 18 in the active phase, and the IFX group, with 30 cases in remission and 15 in the active phase. This study was conducted in accordance with the ethical principles of the Declaration of Helsinki.

The study protocol was approved by the Institutional Review Board/Ethics Committee of the participating center (no. YYLL20240065). Informed consent was obtained from all enrolled patients. In addition, this study is a feasibility study of small samples. The sample size was based on the sample size calculation formula *n* = *Z*^2^ * [*p* * (1 − *p*)]/*δ*^2^, combined with the actual situation of our hospital.

### 2.2 Inclusion and exclusion criteria

The inclusion criteria were: (1) according to the clinical symptoms, laboratory examination, and endoscopic and histological diagnosis of CD; (2) data integrity, with laboratory tests performed after admission related; and (3) adult patients with CD who failed traditional treatments and were subsequently treated with IFX or UST.

The exclusion criteria were: (1) smoking and heavy drinking; (2) abnormal coagulation function; (3) taking antibiotics; (4) with a history of other active autoimmune diseases, malignant tumors, or concurrent chronic diseases such as hypertension and diabetes; and (5) patients who refused treatment with IFX or UST.

### 2.3 Data collection

Patient data including gender, age, comorbidities, surgical history, CDAI scores, C-reactive protein (CRP), hemoglobin (Hb), erythrocyte sedimentation rate (ESR), albumin (ALB), white blood cell (WBC) count, hematocrit (HCT), neutrophil-to-lymphocyte ratio (NLR), neutrophil percentage (N%), lymphocyte ratio (LY%), red blood cell (RBC) count, and lymphocyte (LYM) were collected from the medical system.

### 2.4 Preprocessing of blood samples

Approximately 2 ml of blood was collected from the patient via a venous puncture at the 16 week of treatment and the supernatant obtained through centrifugation. Blood samples were taken out of a −80°C refrigerator and placed in a 4°C refrigerator for thawing. The samples were placed on ice and 100 μl of the sample was collected. Subsequently, 400 μl of an extraction solution (methanol/acetonitrile = 3:1) was added, vortexed for 5 min, and left to stand at 4°C for 2 h. The sample was then taken and centrifuged for 15 min (12, 000 rpm, 4°C), and an equal amount was taken and vacuum concentrated to dryness. Finally, 100 μl of a 50% methanol/water solution (methanol/water = 1:1, *v*/*v*) was added to re-dissolve the sample, vortexed for 3 min (2, 000 rpm, 4°C), centrifuged for 15 min (12, 000 rpm, 4°C), and the supernatant taken for injection analysis.

### 2.5 Quality control

In this study, a strict temperature control and quality monitoring system was established throughout the entire process from sample pretreatment to on-machine testing. During the sample thawing stage, the gradient re-warming method was adopted, maintaining a low-temperature operation on ice throughout to prevent changes in the composition and concentration of metabolic products during the thawing process. In the pretreatment stage of the protein precipitation reagents, the organic solvents used for metabolite extraction were pre-frozen at −20°C to reduce the damage caused by thermally unstable metabolites. Batch quality control strategies were implemented in the HPLC-MS/MS analysis stage. Reagent blanks and quality control (QC) samples were set before and after the analysis of each sample batch to ensure the system stability of the entire detection process.

In the sample detection sequence of this study, an interval insertion strategy was adopted. The QC sample was inserted for every 10 samples to be tested. At the beginning of the sequence, eight QC samples were continuously injected to balance the chromatography–mass spectrometry system and to ensure the stability of the instrument before the formal sample collection began. The retention time shift and the peak area reproducibility of the total ion chromatogram (TIC) or base peak chromatogram (BPC) of the QC samples were the primary indicators for the evaluation of system stability. Ideally, the chromatographic peaks of the QC samples injected at different time points should show a high degree of overlap. In this study, the relative standard deviation (RSD) of the peak area was used as the quantitative threshold for the stability of metabolite quantification. According to the Metabolomics Standards Initiative (MSI) and previous literature, metabolites with RSD ≤30% are considered quantitatively reliable and were retained for subsequent statistical analysis. Metabolites with RSD >30% were excluded due to excessive variation.

### 2.6 Metabolomics analysis

The Q Exactive mass spectrometer (Thermo Fisher Scientific, Waltham, MA, USA) was used to collect the primary and secondary mass spectrometry data. Raw data were imported into the metabolomics processing software for systematic analysis. Core operations such as baseline filtering, characteristic peak identification, peak area integration, retention time calibration, and peak alignment were successively completed, and a multidimensional biological information data matrix containing key dimensions such as the retention time, the mass-to-charge ratio, and the peak intensity was ultimately constructed.

The standardized data were then imported into Metware Cloud, a free online platform, for data analysis. Initially, an unsupervised principal component analysis (PCA) model was employed to assess data stability and examine the original distribution of the two data groups. Subsequently, a supervised orthogonal partial least squares discriminant analysis (OPLS-DA) model was developed to analyze group-related features, with the model validity confirmed through 200 permutation tests. Differential metabolites were identified using the selection criteria of a variable importance in projection (VIP) value greater than 1 and a *p*-value less than 0.05, with *t*-tests conducted to facilitate screening.

### 2.7 Statistical analysis

Data were analyzed using the standard statistical software SPSS 26.0. For categorical variables, the numbers and percentages were reported with absolute 95% confidence intervals, when applicable. For continuous variables, the results were presented as the mean ± standard deviation (SD). When the variable was normally distributed, group differences were analyzed using a *t*-test; otherwise, the Mann–Whitney test was employed. Continuous and categorical variables were tested for statistical significance using a *t*-test, the Wilcoxon rank-sum test, or the chi-square test. A *p-value <0.05 was considered statistically significant, a **p-value <0.01 was considered a highly significant difference, and a ***p-value <0.001 was considered an extremely significant difference.

## 3 Results

### 3.1 Clinical statistics of the study population

#### 3.1.1 Characteristics of the study participants

This study enrolled 81 patients with CD. Based on their intended treatment regimens, the patients were further classified into the UST group, with 18 cases in remission and 18 in the active phase, and the IFX group, with 30 cases in remission and 15 in the active phase. The demographic and clinical baseline characteristics are summarized in **Table 1**. **Table 1** shows that CDAI, CRP, ESR, and ALB demonstrated significant differences (*p* < 0.05) in the baseline characteristics. No statistically significant differences were observed in the other baseline parameters (*p* > 0.05).

**Table 1:** Baseline clinical data sheet.

Variables	Healthy (N=20)	IFX-remission (N=30)	IFX-active (N=15)	UST-remission (N=17)	UST-active (N=18)	P
Gender						0.713
Male	10 (50%)	20 (66.7%)	10 (66.7%)	11 (64.71%)	9 (50.00%)	
Female	10 (50%)	10 (33.3%)	5 (33.3%)	6 (35.29%)	9 (50.00%)	
Age	45.32±3.45	35.45±5.45	34.15±3.35	30.43±5.65	32.45±5.43	0.56
BMI (kg/m2)	23.73±2.29	20.80±3.64	21.88±3.21	21.9±3.48	21.88±3.21	0.11
Disease Duration	–	4.8±2.1	4.0±4.7	6.7±6.9	5.5 ±2.5	0.44
CDAI	–	128±32	254±68	138±28	273±51	0.041
CRP	1.64±0.88	4.48±6.34	11.58±15.9	2.60±1.45	11.58±15.9	0.018
ESR	5.90±4.17	9.10±6.16	17.67±14.08	17.59±14.81	22.28±17.65	0.032
ALB	46.07±3.59	46.92±3.38	44.33±4.35	44.06±4.73	44.33±4.35	0.045
Prior biologic treatment	–	0	0	0	0	–
Steroid treatment	–	0	0	0	0	–
Immunomodulator Treatment	–	1 (3.33%)	0	0	0	–
Surgical history	–	0	1 (6.67%)	0	2 (11.1%)	–
Disease Location						
L1	–	5 (16.6%)	5 (33.3%)	6 (35.3%)	3 (16.7%)	–
L2	–	13 (43.3%)	3 (20.0%)	4 (23.5%)	5 (27.8%)	–
L3	–	9 (30.0%)	7 (46.7%)	5 (29.4%)	8 (44.4%)	–
L4	–	3 (10.0%)	0	2 (11.8%)	2 (11.1%)	–
Disease Behavior						
B1	–	10 (33.3%)	4 (26.7%)	5 (29.4%)	4 (22.2%)	–
B2	–	14 (46.7%)	8 (53.3%)	4 (23.5%)	8 (44.4%)	–
B3	–	4 (13.3%)	3 (20.0%)	7 (41.2%)	6 (33.3%)	–
P	–	2 (6.7%)	0	1 (5.9%)	1 (5.6%)	–

#### 3.1.2 Between-group comparison of the UST remission group and the healthy control group

**Figure 1:**
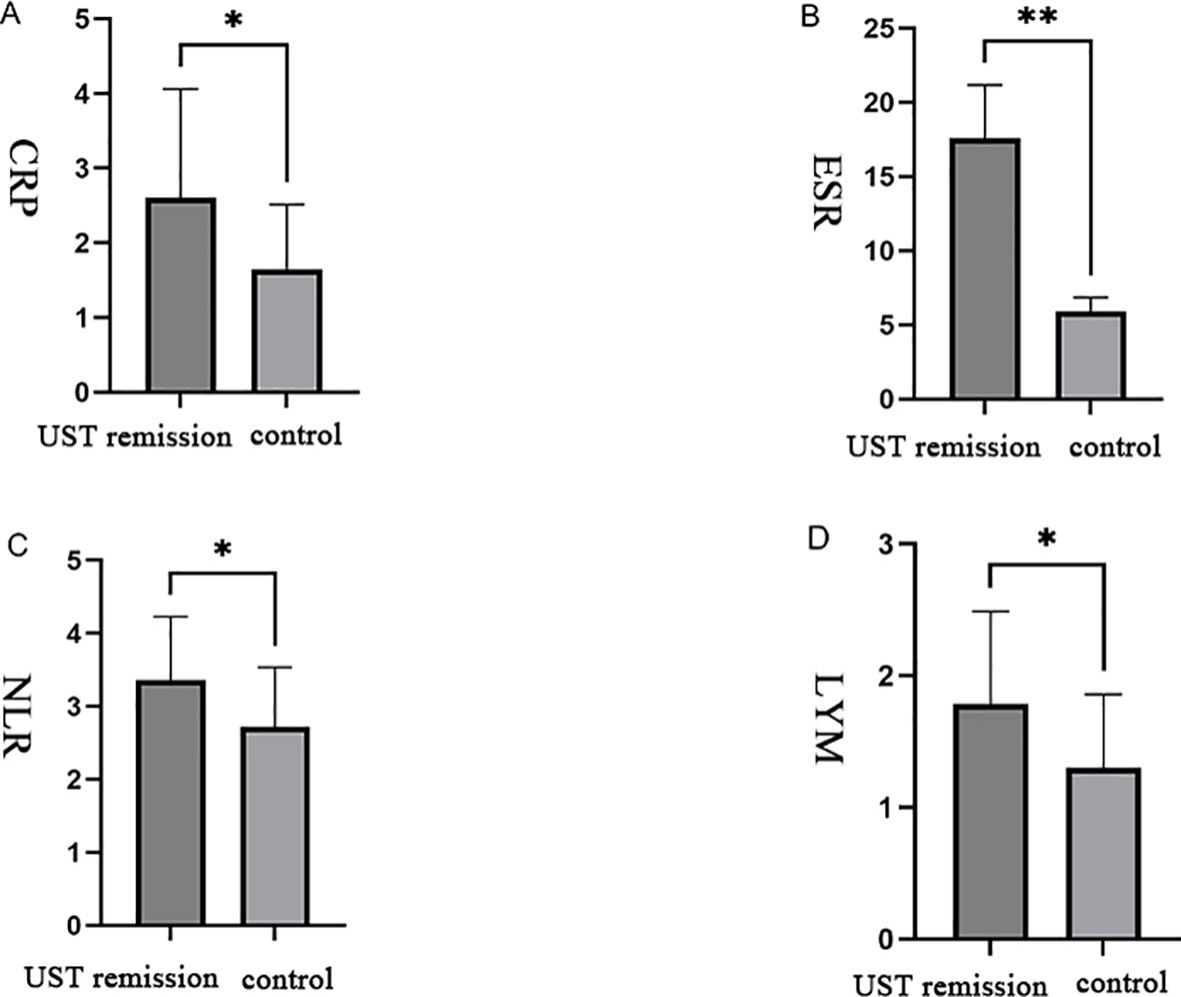
Comparison of the C-reactive protein (CRP), erythrocyte sedimentation rate (ESR), neutrophil-to-lymphocyte ratio (NLR), and lymphocyte (LYM) between the ustekinumab (UST) remission group and healthy controls. ***P<0.001, **p<0.01, *p<0.05.

In this study, we conducted a comparative analysis of the general clinical data between CD patients in remission undergoing UST treatment and a healthy control group. The findings revealed statistically significant differences (*p* < 0.05) in CRP, absolute neutrophil count, NLR, and LYM between the two groups. Notably, the difference in ESR was more pronounced in patients in remission receiving UST treatment compared with healthy individuals (*p* < 0.01), whereas the other indices did not exhibit statistically significant differences (*p* > 0.05), as detailed in **Table 2** and **Figure 1**.

**Table 2:** Between-group comparison of UST remission group and healthy control group ( $\bar{x}$ ± s).

Variables	Remission	Healthy	p
Gender			0.315
Male	11 (64.71%)	10 (50.00%)	
Female	6 (35.29%)	10 (50.00%)	
Age	30.43±5.65	45.32±3.45	0.432
BMI(kg/m2)	21.9±3.48	23.73±2.29	0.372
CRP(mg/L)	2.60±1.45	1.64±0.88	0.026
Hb(g/L)	138.00±25.75	143.40±13.56	0.445
ESR(mm/h)	17.59±14.81	5.90±4.17	<0.01
Alb(g/L)	44.06±4.73	46.08±4.73	0.152
PCT	0.24±0.07	0.23±0.07	0.052
WBC	5.24±1.22	5.11±1.29	0.355
HCT	41.96±6.74	43.26±3.46	0.479
PLT	243.65±83.15	215.65±55.85	0.231
NLR	3.37±0.87	2.72±0.81	0.025
N%	63.38±8.82	61.37±6.16	0.421
LY%	29.26±8.94	29.55±5.81	0.906
LYM	1.79±0.76	1.30±0.56	0.026
RBC	4.87±0.59	4.80±0.39	0.423

#### 3.1.3 Between-group comparison of the UST active group and the healthy control group

**Figure 2:**
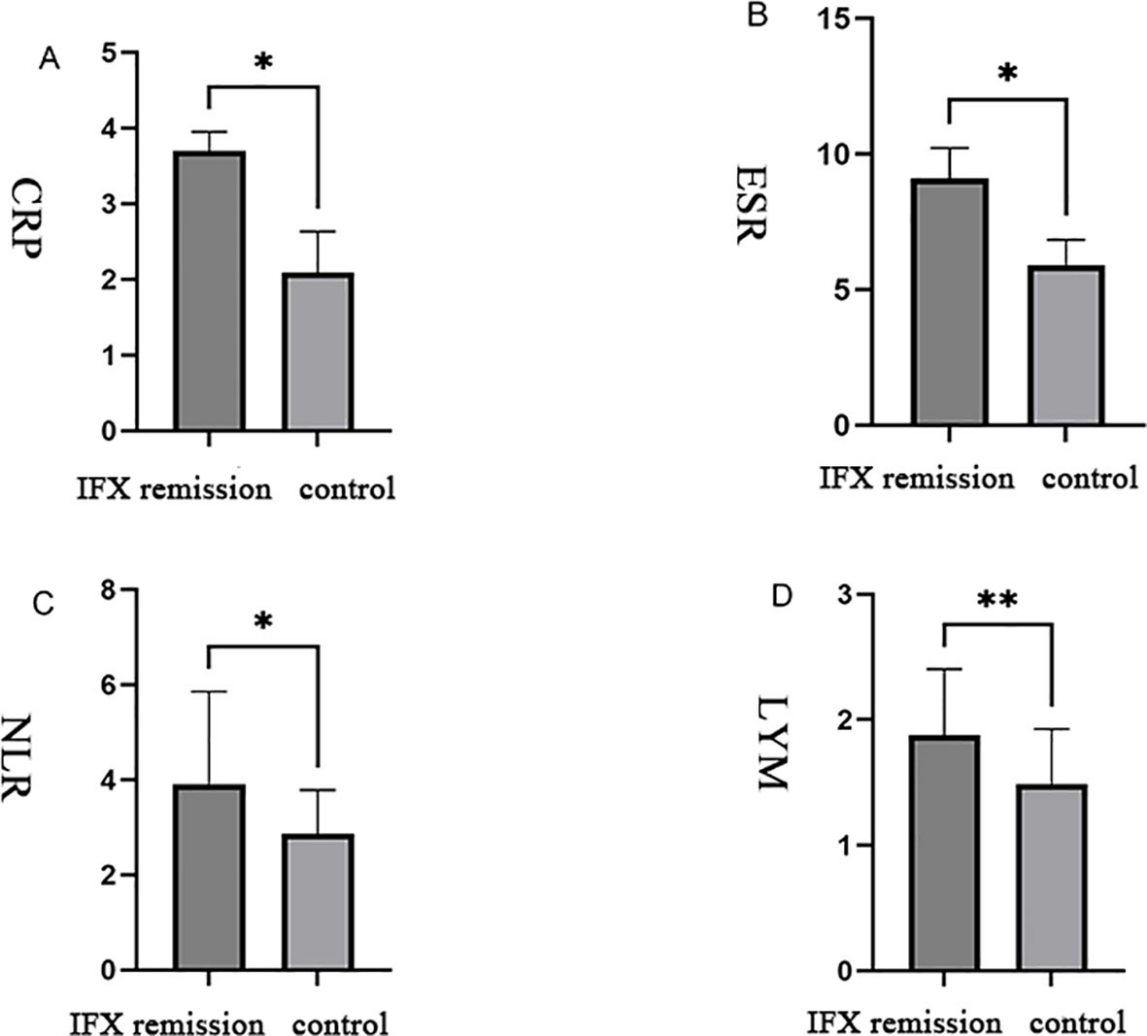
Comparison of the C-reactive protein (CRP), erythrocyte sedimentation rate (ESR), neutrophil-to-lymphocyte ratio (NLR), and lymphocyte (LYM) between the ustekinumab (UST) active group and healthy controls. ***P<0.001, **p<0.01, *p<0.05.

In this segment of the study, we analyzed the general clinical data of patients with active CD undergoing UST treatment in comparison to a healthy control group. The results indicated no significant differences in the general clinical data between patients in the active stage treated with UST and healthy individuals (*p* *>* 0.05). However, significant differences (*p* < 0.05) were observed in the inflammatory indicators, including CRP, WBC, PLT, NLR, and LYM, with the differences in ESR being particularly pronounced (*p* *<* 0.01), as detailed in **Table 3** and **Figure 2**.

**Table 3:** Between-group comparison of UST active group and healthy control group ( $\bar{x}$ ± s).

Variables	Active	Healthy	p
Gender			0.217
Male	9 (50.00%)	10 (50.00%)	
Female	9 (50.00%)	10 (50.00%)	
Age	32.45±5.43	45.32±3.45	0.732
BMI(kg/m2)	21.88±3.21	23.73±2.29	0.083
CRP(mg/L)	11.58±15.9	1.64±0.88	0.017
Hb(g/L)	133.17±23.53	143.40±13.56	0.117
ESR(mm/h)	22.28±17.65	5.90±4.17	<0.01
ALB(g/L)	44.33±4.35	46.07±3.59	0.170
PCT	0.26±0.07	0.23±0.06	0.180
WBC	6.17±1.87	5.10±1.29	0.046
HCT	40.74±6.13	43.26±3.46	0.136
PLT	264.94±80.43	215.65±55.85	0.033
NLR	3.18±1.34	1.30±0.56	0.030
N%	63.38±8.82	61.37±6.16	0.238
LY%	29.26±8.94	29.55±5.81	0.416
LYM	1.94±0.73	1.30±0.56	0.030
RBC	4.77±0.69	4.80±0.39	0.087

#### 3.1.4 Between-group comparison of the IFX remission group and the healthy control group

**Figure 3:**
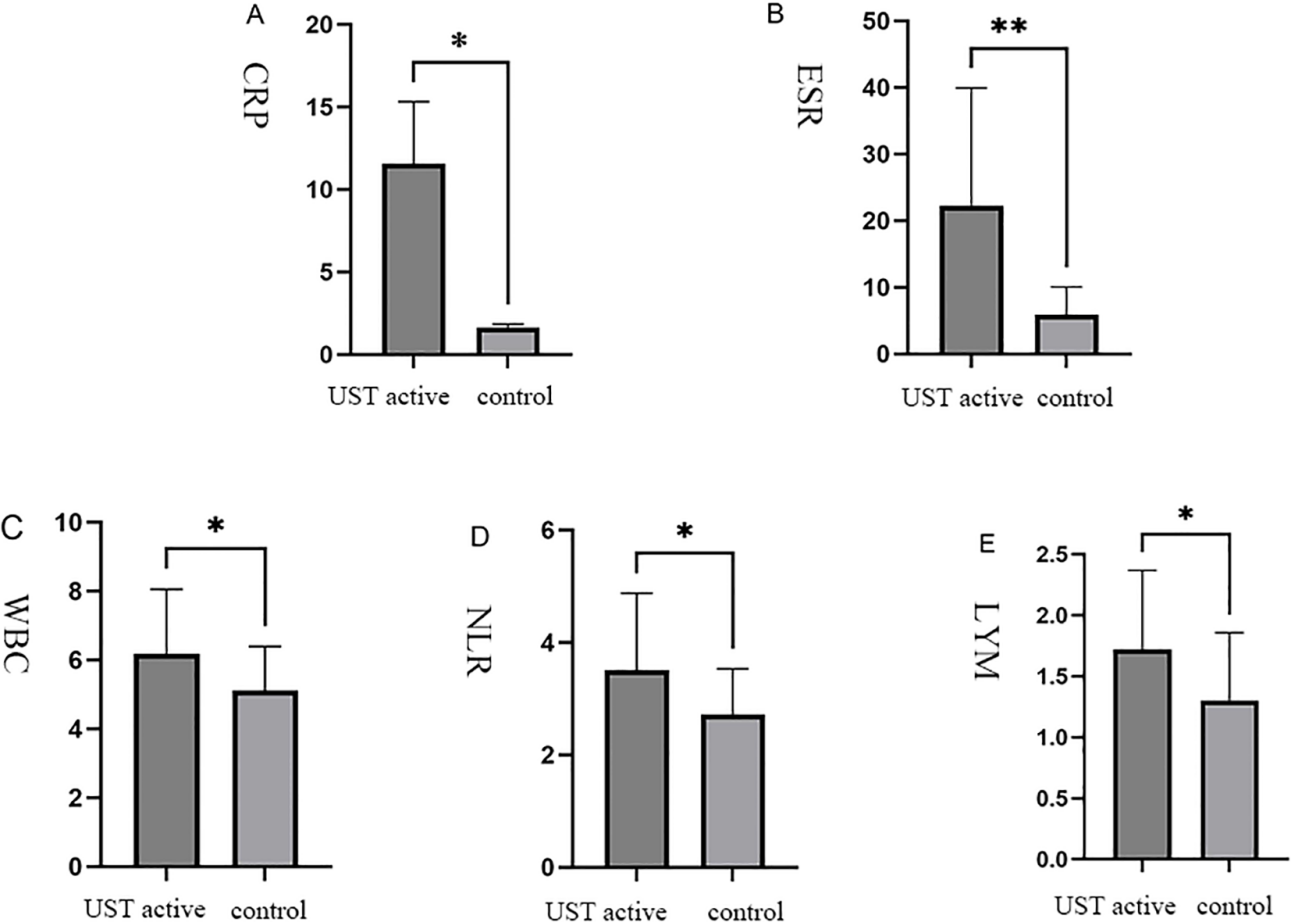
Comparison of the C-reactive protein (CRP), erythrocyte sedimentation rate (ESR), white blood cell (WBC) count, neutrophil-to-lymphocyte ratio (NLR), and lymphocyte (LYM) between the infliximab (IFX) remission group and healthy controls. ***P<0.001, **p<0.01, *p<0.05.

An inter-group comparison of the general clinical data of the patients in the remission phase treated with IFX for CD and the healthy control group indicated no significant differences in gender, age, and body mass index (BMI) between patients in the active stage treated with IFX and healthy individuals (*p* > 0.05). Nevertheless, significant differences (*p* < 0.05) were found in the inflammatory indicators such as CRP, ESR, and NLR, with LYM showing even more significant differences (*p* < 0.01). Detailed results are presented in **Table 4** and **Figure 3**.

**Table 4:** Between-group comparison of IFX remission group and healthy control group ( $\bar{x}$ ± s).

Variables	Remission	Healthy	p
Gender			0.359
Male	20 (66.7%)	10 (50.00%)	
Female	10 (33.3%)	10 (50.00%)	
Age	35.45±5.45	45.32±3.45	0.213
BMI(kg/m2)	20.80±3.64	23.73±2.29	0.102
CRP(mg/L)	4.48±6.34	1.64±0.88	0.045
Hb(g/L)	139.80±16.95	143.40±13.56	0.812
ESR(mm/h)	9.10±6.16	5.90±4.17	0.048
ALB(g/L)	46.92±3.38	46.07±3.59	0.400
PCT	0.26±0.07	0.23±0.06	0.837
WBC	5.35±1.89	5.10±1.29	0.231
HCT	40.74±6.13	43.26±3.46	0.628
PLT	264.94±80.43	215.65±55.85	0.704
NLR	3.51±1.32	2.72±0.81	0.039
N%	63.38±8.82	61.37±6.16	0.077
LY%	29.26±8.94	29.55±5.81	0.061
LYM	1.72±0.65	1.30±0.56	<0.01
RBC	4.86±0.37	4.80±0.39	0.623

#### 3.1.5 Between-group comparison of the IFX active group and the healthy control group

**Figure 4:**
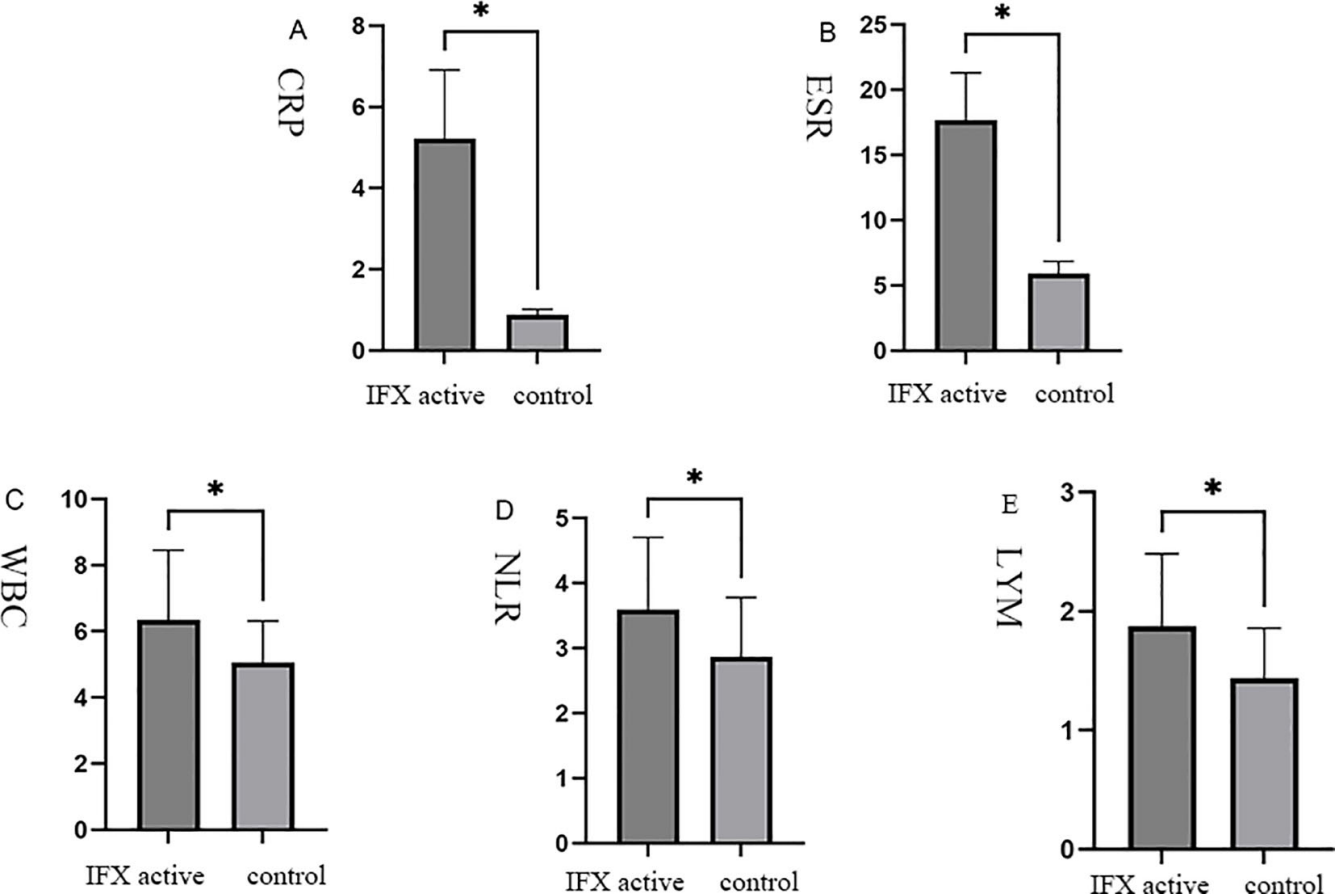
Comparison of the C-reactive protein (CRP), erythrocyte sedimentation rate (ESR), white blood cell (WBC) count, neutrophil-to-lymphocyte ratio (NLR), and lymphocyte (LYM) between the infliximab (IFX) active group and healthy controls. Statistical significance was determined using unpaired two-tailed Student’s *t*-test. ***p < 0.001; **p < 0.01; *p < 0.05. *NS*, not significant.

The analysis focused on general clinical data, which revealed no significant differences in gender, age, and BMI between the patients in the IFX active phase group and the healthy controls (*p* > 0.05). However, significant differences (*p* < 0.05) were observed in the inflammatory markers, including CRP, WBC, NLR, and LYM. Detailed results are presented in **Table 5** and **Figure 4**.

**Table 5:** Between-group comparison of IFX active phase group and healthy control group ( $\bar{x}$ ± s).

Variables	Active	Healthy	p
Gender			0.514
Male	10 (66.7%)	10 (50%)	
Female	5 (33.3%)	10 (50%)	
Age	34.15±3.35	45.32±3.45	0.245
BMI(kg/m2)	21.88±3.21	23.73±2.29	0.271
CRP(mg/L)	11.58±15.9	1.64±0.88	0.026
Hb(g/L)	133.17±23.53	143.40±13.56	0.490
ESR(mm/h)	17.67±14.08	5.90±4.17	0.060
ALB(g/L)	44.33±4.35	46.07±3.59	0.256
PCT	0.22±0.06	0.23±0.06	0.968
WBC	6.34±2.10	5.10±1.29	0.031
HCT	42.20±4.28	43.26±3.46	0.427
PLT	228.27±70.63	215.65±55.85	0.559
NLR	3.59±1.11	2.72±0.81	0.042
N%	63.19±10.26	61.37±6.16	0.516
LY%	26.66±9.02	29.55±5.81	0.258
LYM	2.14±0.88	1.30±0.56	0.016
RBC	4.81±0.45	4.80±0.39	0.215

### 3.2 Metabolomics model validation

#### 3.2.1 Principal component analysis

**Figure 5:**
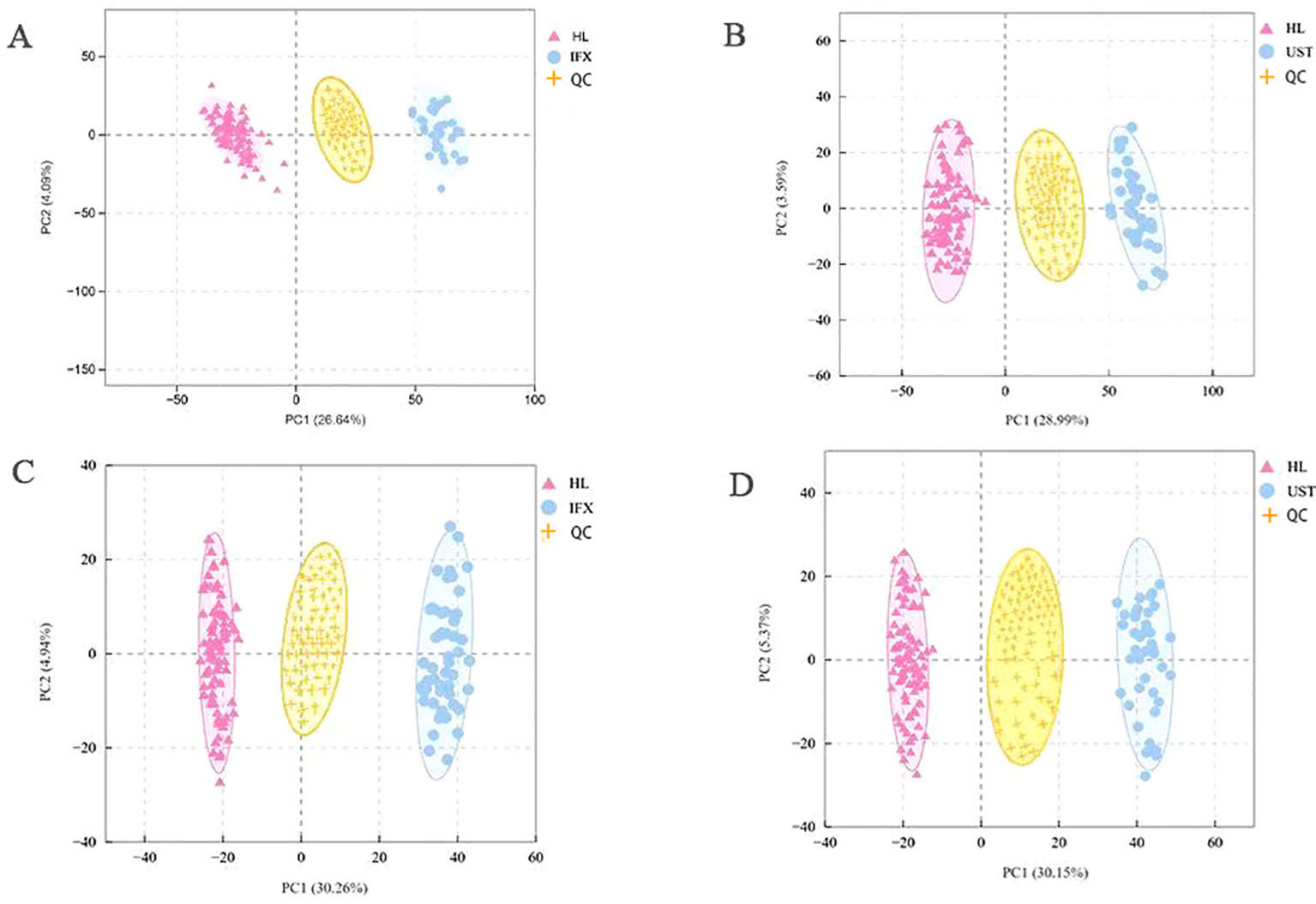
Principal component analysis (PCA) plots. **(A)** PCA of the samples from the healthy control (HL) and infliximab (IFX) groups. PC1 explains 26.64% of the variance, while PC2 explains 4.99%. **(B)** PCA of the samples from the HL and ustekinumab (UST) groups. PC1 explains 3.97% of the variance, while PC2 explains 1.39%. **(C)** PCA of the samples from the HL and IFX groups (replicate/independent experiment). PC1 explains 30.26% of the variance and PC2 explains 4.94%. **(D)** PCA of the samples from the HL and UST groups (replicate/independent experiment). PC1 explains 5.37% of the variance and PC2 explains 5.37%.

We constructed separate PCA models for all ion peaks detected in the positive and negative ion modes (after normalization and Pareto scaling). As shown in **Figure 5**, the PCA score plots for the positive and negative ion modes exhibit highly consistent separation patterns. In both modes, the sample points within the QC sample groups demonstrate good clustering, confirming the stability of the detection method and providing scientific support for the reliability of the data. The original data contained a total of 2, 023 metabolite features. Following filtering based on the RSD in the QC samples (excluding features with an RSD >30%), a total of 210 unstable features were removed, leaving 1, 813 features for subsequent analysis, with a feature retention rate of 89.6%.

#### 3.2.2 OPLS discriminant analysis

**Figure 6:**
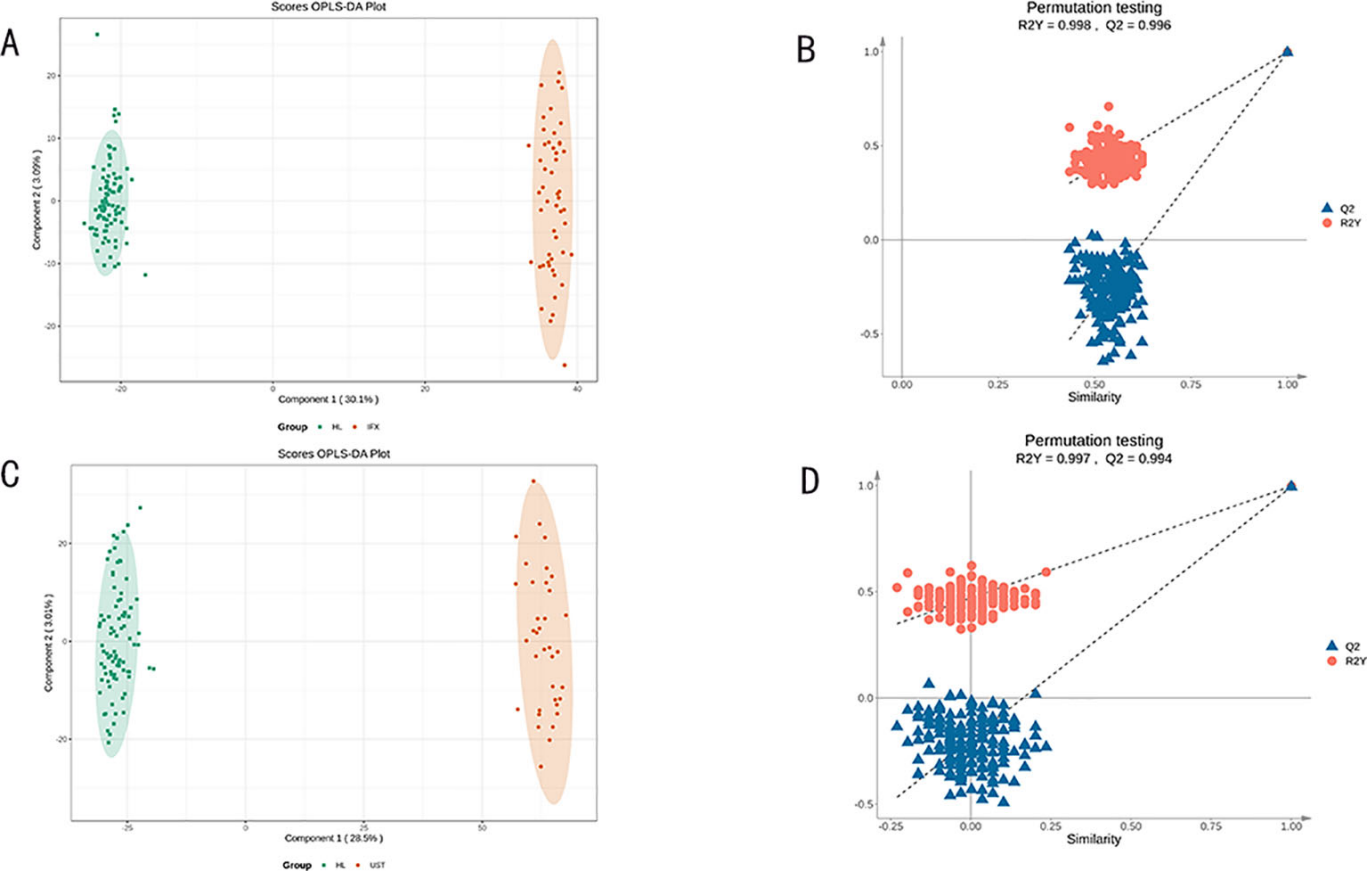
Orthogonal partial least squares discriminant analysis (OPLS-DA) of the samples from the healthy control (HL) and ustekinumab (UST) groups in positive ion mode. **(A)** OPLS-DA score plot showing the separation between the HL and infliximab (IFX) groups. **(B)** Permutation test (200 times) of the OPLS-DA model. **(C)** OPLS-DA score showing the separation between the HL and UST groups. **(D)** Permutation test (200 times) of the OPLS-DA model.

**Figure 7:**
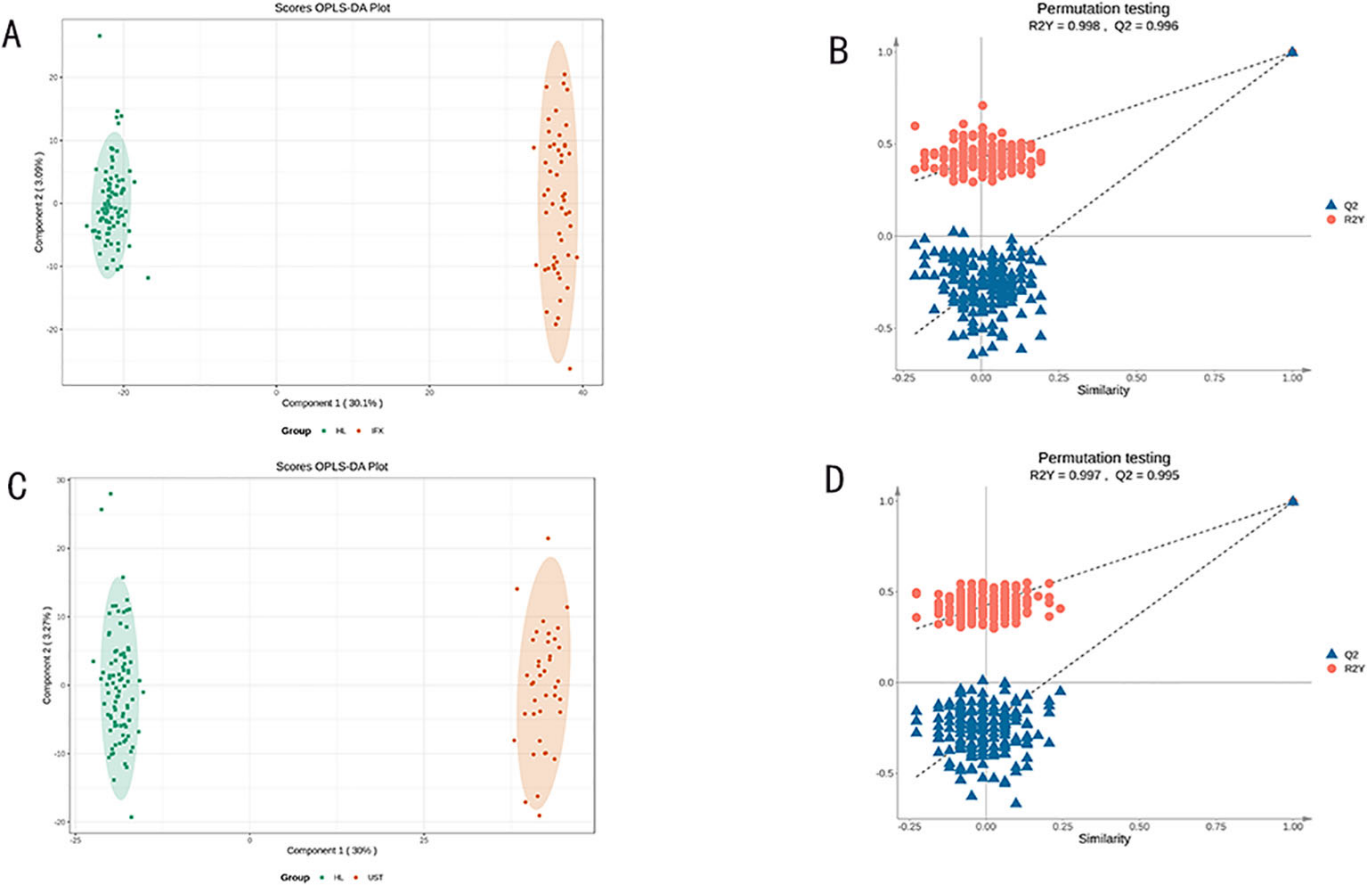
Orthogonal partial least squares discriminant analysis (OPLS-DA) analysis of the samples from the healthy control (HL) and ustekinumab (UST) groups in negative ion mode. **(A)** OPLS-DA score plot showing the separation between the HL and infliximab (IFX) groups. **(B)** Permutation test (200 times) of the OPLS-DA model. **(C)** OPLS-DA score plot showing the separation between the HL and UST groups. **(D)** Permutation test (200 times) of the OPLS-DA model.

The *R*^2^*Y* and *Q*^2^ of the model were calculated using 10-fold cross-validation. The model performance was evaluated with parameters such as *R*^2^*Y* and *Q*^2^, where *R*^2^*Y* reflects the model’s explanatory power for the dependent variable matrix and *Q*^2^ represents the model’s predictive power. The closer the values of these three indicators are to 1, the more stable and reliable the model is. In the OPLS-DA plot, the distribution of the control group and the model group in both positive and negative ion modes showed a trend of intra-group aggregation and inter-group clear separation. To verify the reliability of the OPLS-DA model, 200 permutation tests were conducted. The results showed that the *Q*^2^ values of the real model (positive ion mode: *Q*^2^ = 0.996 and 0.996 for the IFX and UST groups, respectively; negative ion mode: *Q*^2^ = 0.99 and 0.994 for the IFX and in UST groups, respectively) were significantly higher than those of all random permutation models. Moreover, the *Q*^2^ values of the random models had a negative intercept on the *Y*-axis, indicating that the model did not overfit and had excellent predictive ability, as detailed in **Figures 6**, **7**.

### 3.3 Screening of differential metabolites

**Figure 8:**
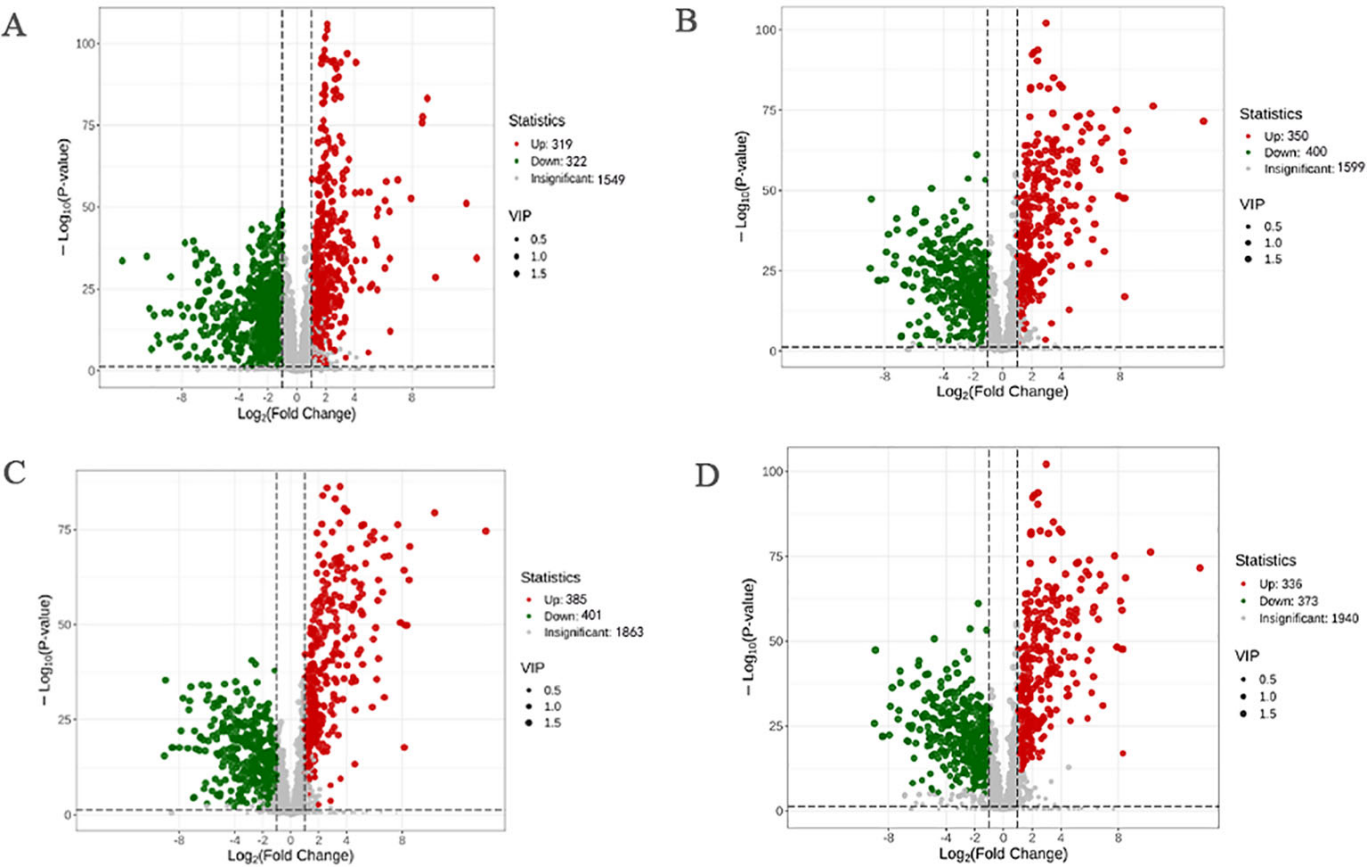
Differential metabolite volcano plot. **(A, B)** Volcano plot for the infliximab (IFX) and ustekinumab (UST) groups, respectively, in cation mode compared with healthy controls (HL). **(C, D)** Volcano plot for the IFX and UST groups, respectively, in cation mode compared with HL.

The screening criteria for differentially expressed metabolites were set as follows: VIP > 1, *p* < 0.05, and *q* < 0.05 after false discovery rate (FDR) correction. In positive ion mode, a total of 641 differentially expressed metabolites were identified in the IFX group, comprising 319 upregulated and 322 downregulated metabolites. In the UST group, a total of 750 differentially expressed metabolites were identified, comprising 350 upregulated and 400 downregulated metabolites. In negative ion mode, a total of 786 differentially expressed metabolites were identified in the IFX group, comprising 385 upregulated and 401 downregulated metabolites. In negative ion mode, a total of 709 differentially expressed metabolites were identified in the UST group, comprising 336 upregulated and 373 downregulated metabolites. Volcano plots were used to illustrate the relative differences in metabolite abundance between the two groups of samples and the statistical significance of these differences. Importantly, the key metabolites identified in this study through metabolic pathway enrichment analysis remained significant after FDR correction, demonstrating their high reliability, as detailed in **Figure 8**.

### 3.4 Screening of biomarkers

**Figure 9:**
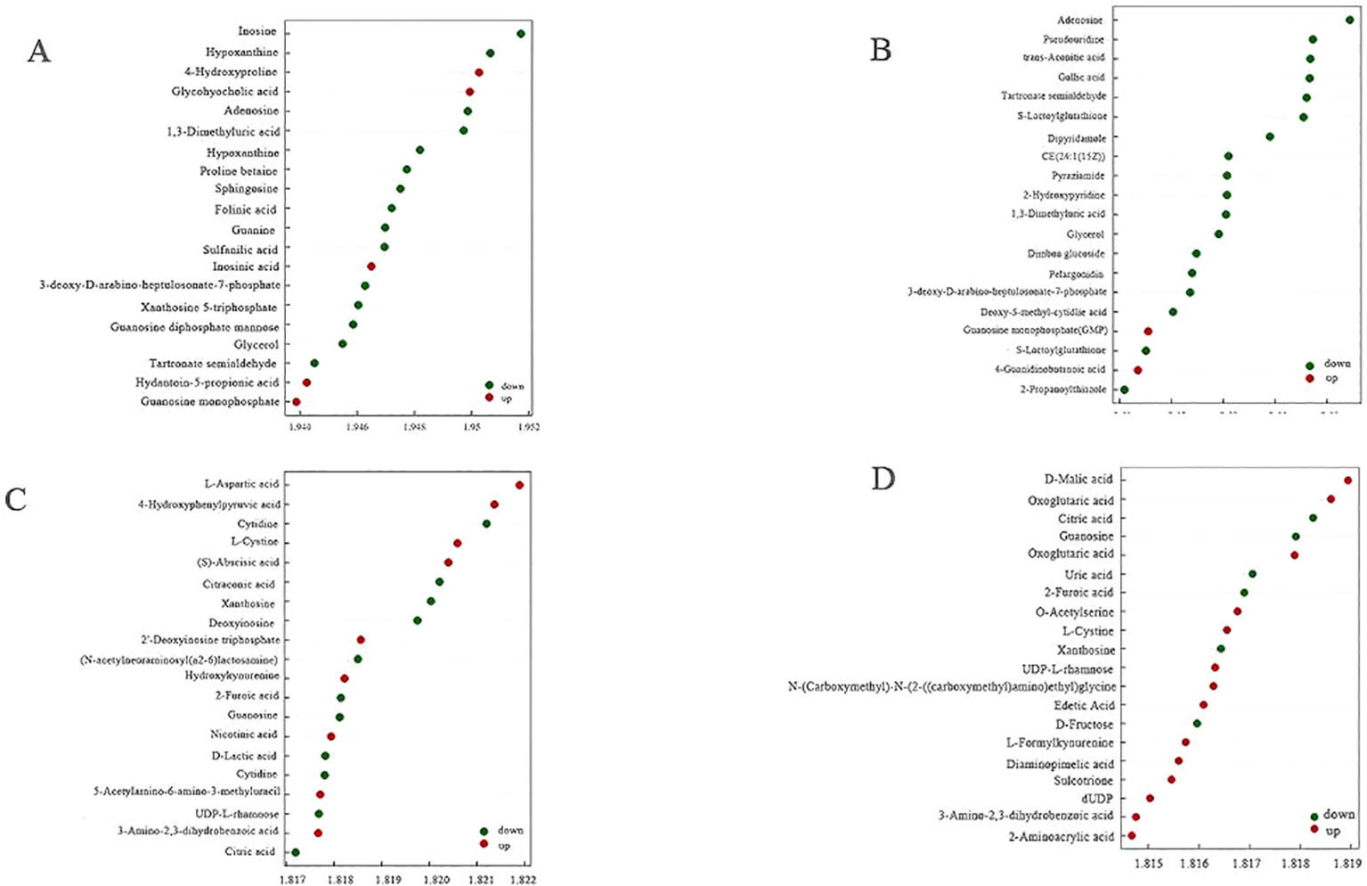
Bubble plot of differential metabolites in the positive and negative ion modes. **(A, B)** show the bubble plot for the IFX and UST groups, respectively, in cation mode, compared with HL. **(C, D)** show the bubble plot for the IFX and UST groups, respectively, in anion mode, compared with HL.

To identify metabolites that exhibit significant inter-group differences, this study established the following thresholds based on multidimensional statistical screening criteria: VIP value (VIP > 1.0), significance level (*p* < 0.05), and fold change (FC ≥ 1.5). Metabolites must satisfy all three criteria simultaneously to be recognized as statistically significant differential metabolites. Ranked according to significance, the top 20 metabolites were selected as potential candidate biomarkers for subsequent in-depth analysis, as detailed in **Figure 9**.** A** total of 20 significantly differentially expressed metabolites were identified in the IFX *vs.* the healthy control group, of which 18 were downregulated and two were upregulated. The top five downregulated metabolites by VIP value were: l-arginine (VIP = 2.34, FC = 0.52, *p* < 0.01), glutathione (VIP = 2.21, FC = 0.48, *p* < 0.01), l-glutamine (VIP = 2.05, FC = 0.61, *p* < 0.01), succinic acid (VIP = 1.98, FC = 0.55, *p* < 0.01), and l-lysine (VIP = 1.87, FC = 0.67, *p* < 0.05). Conversely, the VIP values for the upregulated metabolites were lower, suggesting a comparatively diminished contribution to the differences observed between groups. A total of 20 significantly differentially expressed metabolites were identified in the UST *vs.* the healthy control group, comprising 15 downregulated and five upregulated metabolites. The top three upregulated metabolites by VIP value were: adenosine (VIP = 2.56, FC = 1.85, *p* < 0.01), hypoxanthine (VIP = 2.31, FC = 1.72, *p* < 0.01), and glutathione (VIP = 2.18, FC = 1.68, *p* < 0.01). Among the downregulated metabolites, l-arginine (VIP = 1.92, FC = 0.58, *p* < 0.01) and l-glutamine (VIP = 1.76, FC = 0.63, *p* < 0.05) made significant contributions.

#### 3.4.1 Evaluation of the diagnostic efficacy of metabolic biomarkers

**Figure 10:**
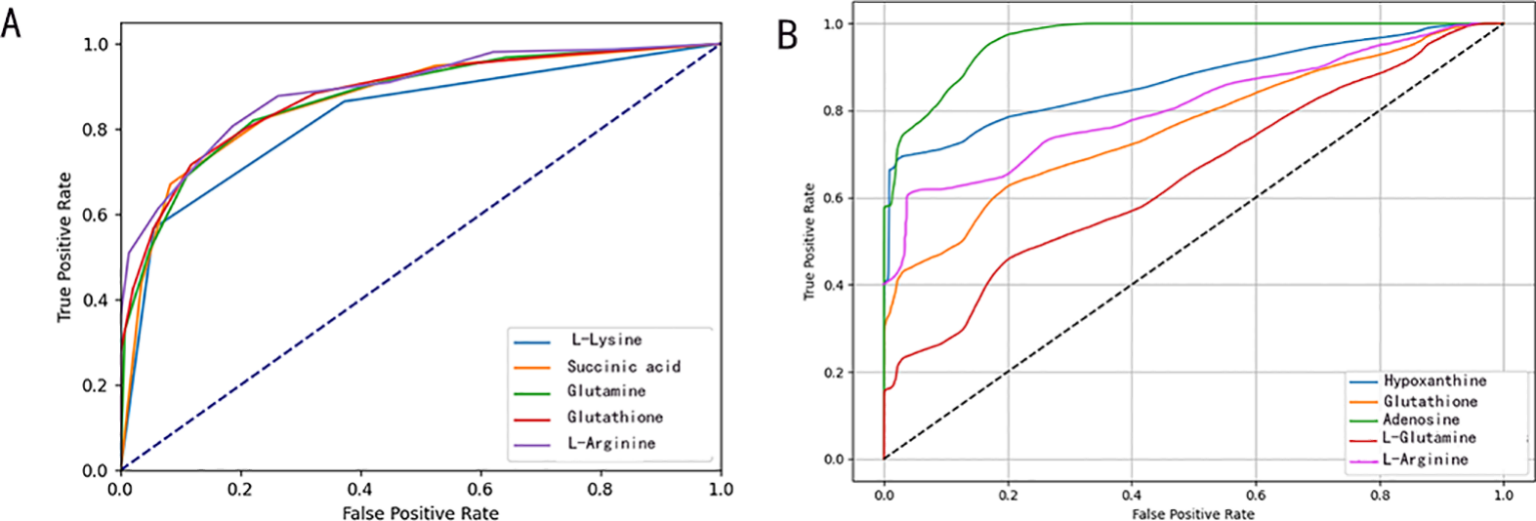
ROC curve for diagnostic markers **(A)** the IFX group **(B)** the UST group.

To assess the diagnostic value of the identified differentially expressed metabolites in distinguishing CD patients from healthy controls, analysis was performed using receiver operating characteristic (ROC) curves. The results showed that l-arginine had an area under the curve (AUC) of 0.89 (95%CI = 0.78–0.94) for distinguishing the IFX group from the healthy controls, with a sensitivity of 82.5% and a specificity of 80.0%, as detailed in **Figure 10**. The AUC for glutathione was 0.88 (95%CI = 0.73–0.91), with a sensitivity of 81.8% and a specificity of 80.2%. The AUC for succinic acid was 0.87, with a sensitivity of 81.3% and a specificity of 79.8%. The AUC for glutamine was 0.87. The AUC for l-lysine was 0.83 (95%CI = 0.73–0.89), with a sensitivity of 78.7% and a specificity of 77.8%. Glutamine had an AUC of 0.87, a sensitivity of 81.0%, and a specificity of 79.8%.

In the comparison between the UST group and healthy controls, hypoxanthine had an AUC of 0.86 (95%CI = 0.77–0.93), with a sensitivity of 81.0% and a specificity of 79.8%. The AUC for glutathione was 0.76 (95%CI = 0.68–0.79), with a sensitivity of 77.5% and a specificity of 77.8%. The AUC for adenosine was 0.96 (95%CI = 0.82–0.96), with a sensitivity of 90.0% and a specificity of 85.9%. The AUC for l-glutamine was 0.68 (95%CI = 0.70–0.79), with a sensitivity of 70.5% and a specificity of 73.8%. The AUC for l-arginine was 0.81 (95%CI = 0.72–0.87), with a sensitivity of 87.0% and a specificity of 85.9%. In summary, the aforementioned biomarkers demonstrated good diagnostic performance.

### 3.5 Enrichment analysis

**Figure 11:**
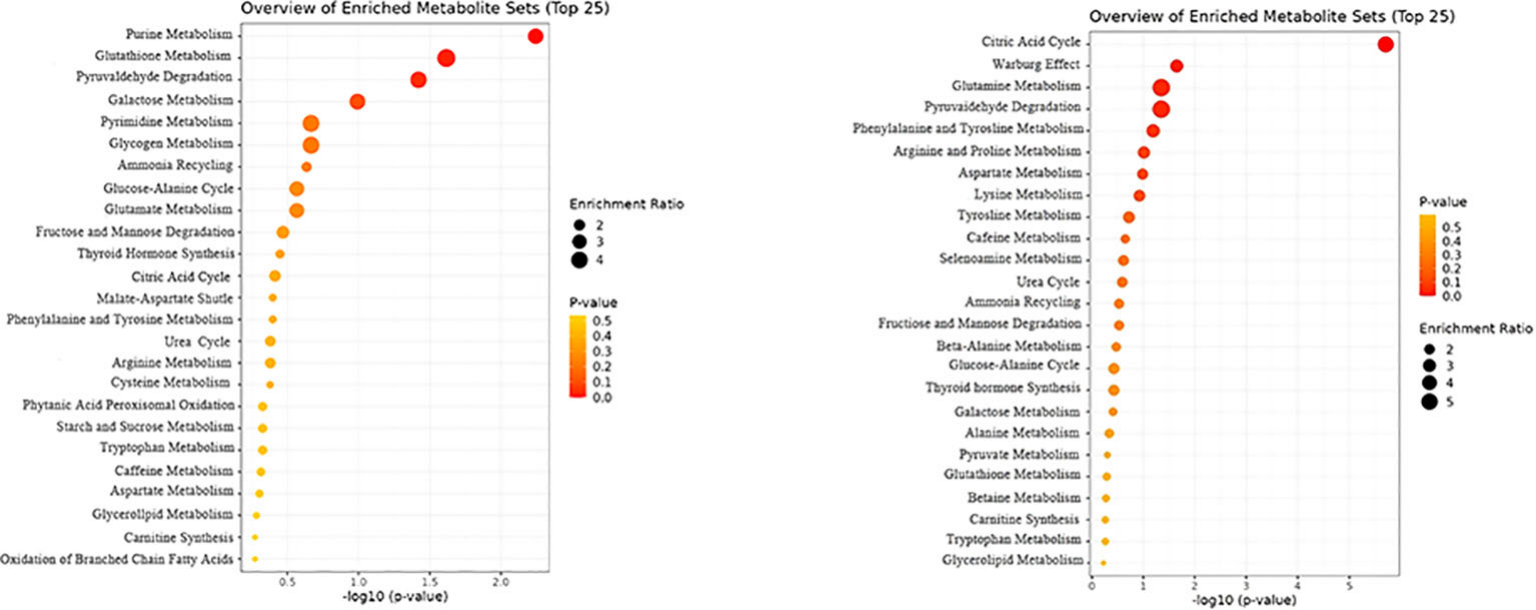
Metabolite enrichment pathway bubble plot.

Enrichment analysis of the metabolic pathways used the MetaboAnalyst 6.0 platform for metabolomics data analysis based on the Kyoto Encyclopedia of Genes and Genomes (KEGG) database, as detailed in **Figure 11**. This platform calculates pathway importance based on topological analysis. Using an FDR threshold of <0.05 and impact >0.1 as the screening criteria, the metabolites in the IFX group were differentially enriched in the following four metabolic pathways: purine metabolism, tricarboxylic acid (TCA) cycle, glyoxylate–dicarboxylic acid metabolism, and cystine–methionine metabolism. The metabolites in the UST group were differentially enriched in the following four metabolic pathways: purine metabolism, alanine, aspartate, glutamate metabolism, and the TCA cycle.

### 3.6 Correlation analysis of the clinical indicators and differential metabolites

**Figure 12:**
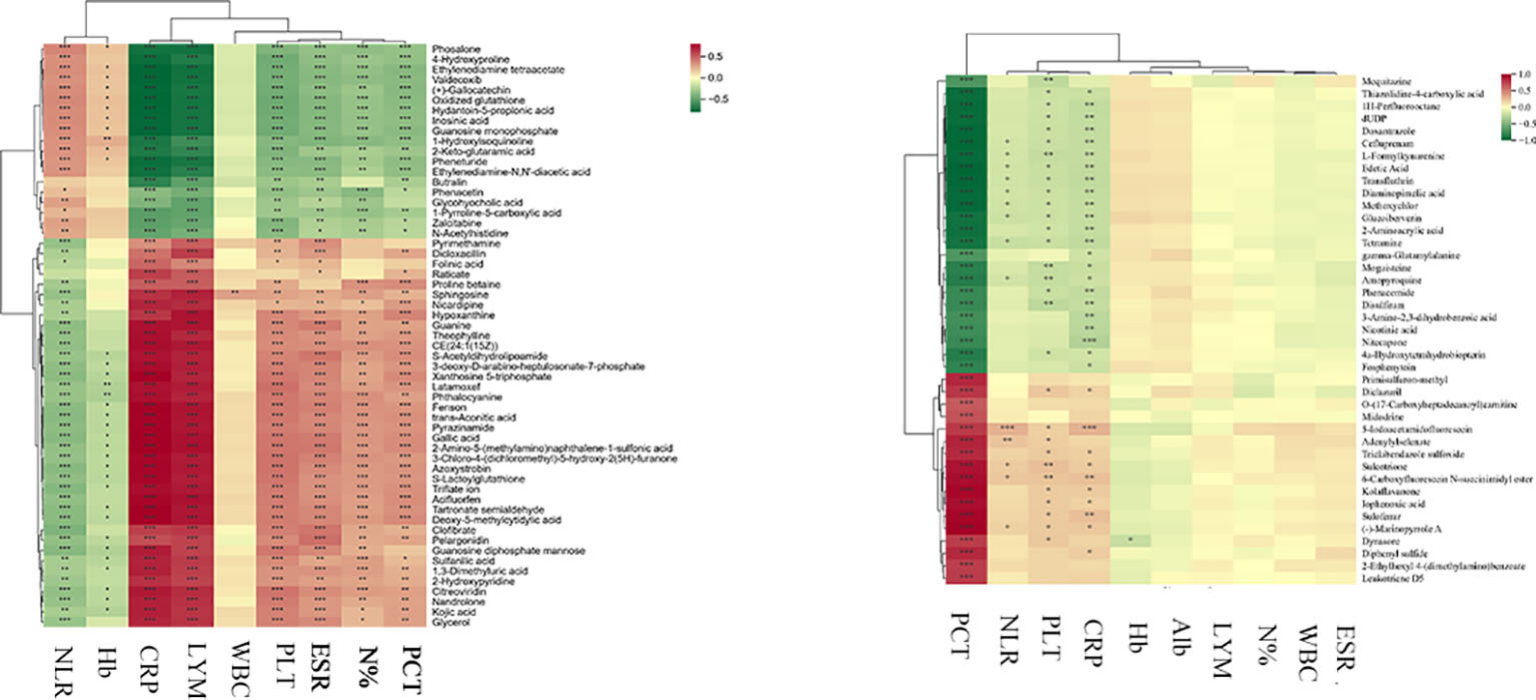
Heatmap of the correlation between the differential metabolites and the clinical indicators.

To delve deeper into the intrinsic relationship between the selected differential metabolites and the clinical phenotypes of the samples, as well as to evaluate their potential clinical application value, Pearson’s correlation analysis was conducted on the significantly different metabolites and key clinical indicators. The results showed that the metabolites in the different treatment groups exhibited characteristic correlation patterns with the clinical indicators. To further explore the relationship between the identified candidate biomarkers and the key inflammatory indicators, Pearson’s correlation analysis was performed, as detailed in **Figure 12**.

The analysis focused on the therapy-specific negative correlations that directly support the proposed biomarker sets. In the IFX treatment group, significant negative correlations were observed between the CRP levels and the concentrations of l-arginine (*r* = −0.52, *p* < 0.01), l-glutamine (*r* = −0.48, *p* < 0.01), and l-lysine (*r* = −0.44, *p* < 0.05). These findings suggest that lower levels of these amino acids are associated with a higher inflammatory burden and that IFX may exert its anti-inflammatory effects partially through restoring the amino acid metabolism. In the UST treatment group, the CRP level and LYM showed significant negative correlations with glutathione (CRP: *r* = −0.46, *p* < 0.01; LYM: *r* = −0.42, *p* < 0.05) and adenosine (CRP: *r* = −0.53, *p* < 0.01; LYM: *r* = −0.49, *p* < 0.01). These findings suggest that elevated concentrations of glutathione and adenosine are correlated with a diminished systemic inflammation and an enhanced immune regulation, aligning with the modulation of purine and glutathione metabolism by UST.

## 4 Discussion

CD is a chronic nonspecific intestinal inflammatory disorder with an unclear cause. Currently, biologic agents have become an indispensable and important means in the clinical treatment of CD. Among them, IFX and UST are two representative types of drugs widely used in clinical practice. IFX is an antitumor necrosis factor preparation, while UST is an anti-IL-12/IL-23 preparation. Although both types of drugs have shown certain clinical efficacy in the treatment of CD, there are significant individual differences in the responses of different patients to IFX and UST, and their effects on the overall metabolic status of the patients have not been fully clarified.

HPLC-MS/MS technology, with its advantages of high sensitivity, high resolution, and wide detection range, has been widely applied in metabolomics research related to immune diseases. It can precisely identify subtle changes in the metabolic profile of the body. In this study, HPLC-MS/MS technology was used to analyze differences in the metabolites between CD patients treated with IFX and UST at week 16, with a view to identifying biomarkers for early diagnosis.

### 4.1 Effects of infliximab and usulizumab on blood metabolites

This study found that, regardless of the IFX or UST treatment group, the inflammatory indicators of both patient groups in the remission and active phases were significantly higher than those of the healthy control group (*p* < 0.05). The UST patients in the remission phase still showed significant differences in indicators such as ESR and CRP compared with the healthy control group (*p* < 0.05), while the IFX patients in the remission phase exhibited stronger abnormalities in the absolute value of lymphocytes. This finding reveals that IFX and UST have different characteristics and limitations in controlling inflammation and immune regulation. The reasons may be as follows:

1. CD is a chronic and recurrent immune dysregulation disease. Even when clinical remission is achieved, the intestinal mucosa may still have microscopic visible low-level inflammation and that the immune cascade reaction has not been completely terminated (15, 16).
2. The differences in their mechanism of action may have led to different abnormal patterns of the inflammatory indicators in the IFX and UST treatment groups (17, 18). The research results of Torres et al. (19) showed that, in patients with CD who achieved mucosal healing after UST induction therapy, the mucosal gene expression profiles still demonstrated significant differences from those of the healthy control group. This result indicates that clinical remission does not equate to biological or histological healing. Even if the histological inflammation subsides, molecular-level inflammatory abnormalities still persist, and the immune cascade reaction may still be ongoing at a low level.

This study found that the CRP and ESR of the patients in the UST remission period were still higher than those of the healthy control group. This may be due to the following:

1. Although the IL-12/23 pathway is blocked, differentiated effector T cells might continue to produce pro-inflammatory factors such as IFN-γ. Other pro-inflammatory cytokines such as IL-6 might maintain low-level inflammation through alternative pathways, while the synthesis of CRP is mainly regulated by IL-6. Corcoran’s *in vitro* study confirmed that, although UST treatment could significantly reduce IL-23 secretion (*p* = 0.043), the reduction degree of IL-6 and MCP-1, among other chemokines, varied between individuals (20).
2. Tissue-resident memory (TRM) T cells would persist in incompletely healed mucosal tissues. This local, low-level inflammation, although not sufficient to cause clinical symptoms, is sufficient to drive a mild increase in systemic inflammatory markers. A study (21) found that, despite the administration of anti-IL-12/IL-23 biologic agents, residual CD103^+^ TRM T cells might still be present in local tissues. These TRM cells reside in tissues and, once activated by local antigens, can rapidly produce IFN-γ and IL-22, among other pro-inflammatory factors, driving local low-level inflammation, thereby causing a persistent mild increase in systemic inflammatory markers.

The mechanism of action of IFX is not limited to neutralizing soluble and transmembrane TNF-α but also involves inducing the apoptosis of activated T lymphocytes. This mechanism is crucial for the control of intestinal inflammation, but may also lead to changes in the distribution of LYM subgroups, resulting in abnormal absolute values of lymphocytes in IFX remission period patients. Schnell et al. (22) found that, after 12 months of IFX treatment, the transitional B cells that produce IL-10 in responders significantly increased. The upregulation of regulatory B cells may be a compensatory response of the body to the inflammatory state, but may also lead to complex changes in the overall lymphocyte count. The clinical indicator analysis results of this study showed that the inflammatory markers of the patients treated with IFX were significantly different from those of the control group (*p* < 0.05). However, the serum ALB levels of the patients were not significantly different from those of the control group (*p* > 0.05), indicating that IFX can effectively regulate the inflammatory response of patients with CD, but has no significant improvement effect on the serum ALB levels related to protein synthesis. It is speculated that IFX cannot prevent the continuous loss of nutritional substrates, which may be related to its inability to improve the absorption dysfunction of the intestinal mucosa in patients with CD. The results of this nutritional study are consistent with those of a previous research of the group. Enteral nutrition (EN) can be used as an auxiliary nutritional treatment for patients with CD to improve their nutritional absorption defects. Therefore, for CD patients with poor nutritional indicators, especially those with moderate to severe active stage, this clinical treatment can use biologic agents combined with EN. This clinical plan also complements the results of this study and provides an important theoretical basis for the subsequent combined application of IFX+EN in the treatment of CD.

### 4.2 Effects of IFX and UST on blood metabolites

This study, using non-targeted metabolomics analysis, found that IFX and UST treatment exerted significantly different effects on the blood metabolites in patients with CD: IFX primarily enriched the metabolic pathways related to energy metabolism, such as amino acid metabolism, glycolysis, and the TCA cycle, whereas UST treatment mainly affected the pathways related to glucose metabolism, glutathione metabolism, and purine metabolism. This suggests that the anti-inflammatory effects of the two biologics are closely associated with the regulation of energy metabolism, the enhancement of antioxidant capacity, and the influence on nucleic acid synthesis. This difference may be attributable to the distinct mechanisms of action of the two biologics.

As an anti-TNF-α monoclonal antibody, IFX mediates its anti-inflammatory effects through the neutralization of both soluble and transmembrane forms of TNF-α and by the induction of apoptosis in activated T lymphocytes. This mechanism necessitates the swift activation and subsequent clearance of immune cells, thereby imposing substantial demands on energy metabolism. When activated T cells transition from a resting state to an effector state, their metabolic profile shifts from oxidative phosphorylation to glycolysis and glutamine breakdown to meet biosynthetic demands. TNF-α stimulation leads to increased TCA cycle metabolites and significantly influences the expression of the proteins associated with amino acid, energy, and nucleotide metabolism (23). Souza et al. (24) found that TNF-α primarily utilizes alanine metabolism for energy production via the TNFR1 signaling pathway. Guo et al. (25) found that patients in the remission group exhibited significantly elevated levels of amino acid metabolites such as l-proline and histidine at baseline, indicating that amino acid metabolic pathways play a crucial role in the response to IFX treatment. Consequently, IFX mediates the inhibition of TNF-α signaling. The energy metabolic demands driven by inflammation are alleviated, leading to alterations in the energy metabolic pathways such as amino acid metabolism, glycolysis, and the TCA cycle (26). This also explains the fundamental reason for the enrichment of IFX in these pathways in the present study. A clinical study by Nishida et al. (27) also confirmed that respiratory entropy in patients with CD increased significantly following anti-TNF-α treatment, indicating a shift in energy metabolism from lipid oxidation to glucose utilization.

UST inhibits Th1 and Th17 cell differentiation by targeting the IL-12/IL-23 p40 subunit, with its anti-inflammatory action focusing more on blocking the initial stages of the inflammatory cascade rather than directly eliminating the already activated effector cells. Its anti-inflammatory mechanism differs fundamentally from that of IFX. This study found that UST primarily influences glutathione metabolism, a pathway that constitutes the body’s most important antioxidant defense system. The glutathione synthesis pathway is positively correlated with the levels of circulating pro-inflammatory cytokines in patients with active CD, and the glutathione levels in the blood increase in response to persistent oxidative stress within the body (28). Activation of the IL-23/Th17 axis can induce the production of reactive oxygen species, leading to oxidative stress damage by blocking this pathway. UST may indirectly enhance the body’s antioxidant capacity, thereby altering glutathione metabolism. Furthermore, this study also found that purine metabolism plays a significant role in the development of CD inflammation. The IFN-γ secreted by Th1 cells promotes the release of ATP from intestinal epithelial cells or immune cells, further exacerbating the imbalance in purine metabolism. By inhibiting IL-12/IL-23 to reduce the IFN-γ levels in CD patients, UST alleviates intestinal inflammation while helping to restore the pro-inflammatory and anti-inflammatory balance in purine metabolism. Improvements in purine metabolism, in turn, suppress the excessive activation of immune cells, thereby forming a closed anti-inflammatory loop. This is consistent with the mechanism of action of UST and explains the core rationale for its use in the treatment of CD.

### 4.3 Correlation between the metabolites and clinical indicators suggests the existence of potential biomarkers

By integrating non-target blood metabolomics data with laboratory test indicators, this study further revealed the intrinsic connection between molecular phenotypes and macroscopic clinical phenotypes in the pathological process of CD. The research found a significant correlation between the disruption of specific metabolic pathways and the level of systemic inflammation, providing a more systematic perspective for understanding the disease state and the mechanism of drug action. Firstly, metabolic reprogramming centered on amino acid metabolism and energy metabolism is closely related to the disease activity.

In this study, it was observed that l-arginine, l-glutamine, and l-lysine in the IFX treatment group were negatively correlated with the inflammatory marker CRP, and these amino acids play a crucial role in immune regulation. l-Arginine serves as a precursor substance in various metabolic pathways, especially in the synthesis of nitric oxide, which is essential for the maintenance of vascular endothelial function and immune regulation (29). Under inflammatory conditions, the availability of l-arginine limits the activation of macrophages and T lymphocytes, thereby affecting the immune response (30). l-Glutamine, as a conditionally essential amino acid, plays an important role in stress and catabolic processes. Moura et al. (31) demonstrated that l-glutamine can enhance the tolerance of the immune system by regulating the expression of heat shock proteins and restore the activity of antioxidant enzymes during intense exercise. l-Glutamine has also shown significant effects in regulating inflammatory characteristics, reducing the production of inflammatory factors, and increasing the expression of anti-inflammatory factors (32). In addition, l-lysine also plays an important role in immune regulation. l-Lysine can alleviate the damage of oxidative stress to proteins, thereby maintaining protein function (33). These amino acids contribute to immune system functionality by directly engaging in metabolic pathways and augmenting the body’s immune capacity through the regulation of the inflammatory responses and antioxidant defense mechanisms. In the context of chronic inflammatory conditions, persistently activated immune cells can result in the excessive consumption or metabolic imbalance of these essential amino acids. Consequently, it is hypothesized that the therapeutic efficacy of IFX may not only stem from its direct neutralization of the inflammatory mediators but also from its potential to remodel the metabolic patterns of immune cells, thereby mitigating the inflammatory response.

Furthermore, studies have found that the increase in oxidative stress may be related to the imbalance of purine metabolites (34). The disorder of purine metabolism leads to an enhancement of oxidative stress, thereby intensifying the inflammatory response (35). The imbalance of purine metabolism is not only related to oxidative stress but may also affect inflammatory markers such as the CRP and LYM levels. Vuerich et al. (36) found that the adenine signaling pathway in the intestinal mucosa was dysregulated. Adenine, as an intermediate product of purine metabolism, could inhibit the inflammatory response by binding to receptors on the surface of immune cells. However, in patients with CD, the abnormal increase in adenosine deaminase activity in the intestine leads to the accelerated degradation of adenosine, weakening the anti-inflammatory effect and thereby exacerbating the pro-inflammatory differentiation of Th1/Th17 cells. These results indicate that, although UST can induce clinical remission in patients with active CD and enable routine indicators to return to normal, it fails to completely reverse the deep-seated inflammatory response. We speculate that UST can significantly inhibit the release and transmission of pro-inflammatory cytokines by efficiently blocking the IL-12/IL-23 signaling axis, thereby reducing the clinical inflammatory-related indicators in patients with CD. However, as CD is a chronic and recurrent intestinal inflammatory disease, its pathological process may be accompanied by a persistent redox imbalance state. This characteristic leads to the presence of low-level inflammation in the local and systemic areas of patients even when they enter the clinical remission stage, making it difficult to achieve complete inflammation resolution.

## 5 Conclusion

Patients with CD who have attained clinical remission following treatment with IFX and UST may still experience underlying inflammation. These therapeutic agents exert their anti-inflammatory effects via distinct metabolic pathways: IFX predominantly influences amino acid and energy metabolism, while UST targets glucose, glutathione, and purine/pyrimidine metabolism.

## Data Availability

The raw data supporting the conclusions of this article will be made available by the authors, without undue reservation.
