# Supplementary material for: Clinical study on the screening of biomarkers for Crohn’s disease based on blood metabolomics
Source: Front Gastroenterol (Lausanne). 2026 Jul 23;5:1867424. doi: 10.3389/fgstr.2026.1867424 (PMC13441770; doi:10.3389/fgstr.2026.1867424)
Supplement: Supplementary file 1 [file Table1.docx]

**Supplementary Data Table 1 CDAI Scoring Criteria**

| Variable | Weighting |
| --- | --- |
| Frequency of loose stools (per week) | 2 |
| Severity of abdominal pain (overall score for the week, 0–3 points) | 5 |
| General condition (overall score for the week, 0–4 points) | 7 |
| Extraintestinal manifestations and complications (1 point per item) | 20 |
| Opioid anti-diarrhoeal medicines (0–1 marks) | 30 |
| Abdominal mass (suspicious: 2 points; definite: 5 points) | 10 |
| Reduced haematocrit | 6 |
| 100 × (1 – (body weight / standard body weight)) | 1 |

Note: The CDAI score assesses the severity of disease activity. A CDAI score of <150 indicates remission; a score of ≥150 indicates active disease; scores between 150 and 200 are classified as mild; scores between 221 and 450 are classified as moderate; and scores >450 are classified as severe.

**Supplementary Data Table 2 Simplified Endoscopic Score for Crohn’s Disease (SES-CD)**

| Assessment criteria | Weighting |
| --- | --- |
| Ulcer area | 0 points: no ulcers, 1 point: (0.1–0.5 cm in diameter), 2 points: larger ulcers |
| Percentage of the intestine affected by ulcers | 0 points: no ulcers, 1 point: <10%; 2 points: 10%–30%, 3 points: >30% |
|  |  |
| Percentage of the bowel segment affected | 0 points: no lesions, 1 point: <50%, 2 points: 50%–75%, 3 points: >75% |
| Is there any narrowing | 0 points: No stenosis,1 point: A single stenosis, but the vessel is patent, 2 points: Multiple stenoses, but the vessel is patent, 3 points: The vessel is occluded |
| Total score | Total score for the five intestinal segments |

Note: The five intestinal segments include the rectum, the descending and sigmoid colon, the transverse colon (including the splenic flexure), the ascending colon (including the ileocecal region and the hepatic flexure), and the ileum: Assessment of each intestinal segment must be conducted over a length of at least 10 cm to be considered a valid examination. Cases where the endoscope cannot pass through due to surgical resection or intestinal stricture, or where the assessment is deemed invalid due to endoscopic manoeuvring techniques, are excluded. Currently, a total score of 0–2 indicates remission, 3–6 indicates mild activity, 7–15 indicates moderate activity, and ≥16 indicates severe activity.
